# Timeliness and completeness of routine childhood vaccinations in young children residing in a district with recurrent vaccine-preventable disease outbreaks, Jerusalem, Israel

**DOI:** 10.2807/1560-7917.ES.2019.24.6.1800004

**Published:** 2019-02-07

**Authors:** Chen Stein-Zamir, Avi Israeli

**Affiliations:** 1 The Hebrew University of Jerusalem, Faculty of medicine, the Hebrew University and Hadassah Braun School of Public health and Community Medicine, Jerusalem, Israel; 2 Jerusalem District Health Office, Ministry of Health, Jerusalem, Israel; 3 The Hebrew University of Jerusalem, Faculty of medicine, the Hebrew University and Hadassah Braun School of Public health and Community Medicine, Department of Health Policy and Management, Jerusalem, Israel; 4 Chief Scientist, Ministry of Health, Jerusalem, Israel

**Keywords:** vaccines and immunisation, routine vaccinations, children, infants, toddlers, vaccination timeliness, vaccination completeness, vaccination coverage, measles, childhood vaccination, vaccination schedules

## Abstract

**Background:**

Childhood vaccination schedules recommend vaccine doses at predefined ages.

**Aim:**

We evaluated vaccination completeness and timeliness in Jerusalem, a district with recurrent vaccine-preventable disease outbreaks.

**Methods:**

Vaccination coverage was monitored by the up-to-date method (vaccination completeness at age 2 years). Timeliness of vaccination was assessed in children (n = 3,098, born in 2009, followed to age 48 months, re-evaluated at age 7 years) by the age-appropriate method (vaccine dose timeliness according to recommended schedule). Vaccines included: hepatitis B (HBV: birth, 1 month and 6 months); diphtheria, tetanus, acellular pertussis, polio, *Haemophilus influenzae* b (DTaP-IPV-Hib: 2, 4, 6 and 12 months); pneumococcal conjugate (PCV: 2, 4 and 12 months); measles-mumps-rubella/measles-mumps-rubella-varicella (MMR/MMRV: 12 months) and hepatitis A (HAV: 18 and 24 months).

**Results:**

Overall vaccination coverage (2014 cohort evaluated at age 2 years) was 95% and 86% for MMR/MMRV and DTaP-IPV-Hib4, respectively. Most children (94%, 91%, 79%, 95%, 92% and 82%) were up-to-date for HBV3, DTaP-IPV-Hib4, PCV3, MMR/MMRV1, HAV1 and HAV2 vaccines at 48 months, but only 32%, 28%, 38%, 58%, 49% and 20% were vaccinated timely (age-appropriate). At age 7 years, the median increase in vaccination coverage was 2.4%. Vaccination delay was associated with: high birth order, ethnicity (higher among Jews vs Arabs), birth in winter, delayed acceptance of first dose of DTaP-IPV-Hib and multiple-dose vaccines (vs MMR/MMRV). Jewish ultra-Orthodox communities had low vaccination coverage.

**Conclusions:**

Considerable vaccination delay should be addressed within the vaccine hesitancy spectrum. Delays may induce susceptibility to vaccine-preventable disease outbreaks; tailored programmes to improve timeliness are required.

## Background

Vaccines have contributed to substantial reductions of morbidity and mortality from vaccine-preventable diseases (VPD), mainly in children. Vaccinations avert 2–3 million deaths annually; if global vaccination coverage improves, another 1.5 million deaths are preventable [1]. The estimated global coverage for the first dose of measles-containing vaccine (MCV1) and for diphtheria-tetanus-pertussis (DTP3) was 82% for both in 2009 and 85% and 86%, respectively, in 2014; this is below the Global Vaccine Action Plan (GVAP) targets of 90% nationally and 80% in all districts [2]. For measles, the recommended coverage is higher, at 95% or more across all districts and age groups [3].

Routine vaccinations in Israel are included in the National Health Insurance Law. Community-based maternal and child health (MCH) clinics provide free vaccination to children regardless of civil status, with high rates (96%) of service utilisation [4,5]. Vaccine doses are documented in digital health files. The overall vaccination coverage rates reported in Israel are adequate (at age 2 years in 2016: DTaP-IPV-Hib4 at 94%, HBV3 at 97% and MMR/MMRV1 at 96%) with all districts well in line with World Health Organization (WHO) goals [6,7]. Yet, VPD outbreaks observed in specific communities (Arab Bedouin in southern Israel and Jewish ultra-Orthodox in Jerusalem) revealed under-immunised population groups [8-10].

**Figure 1:**
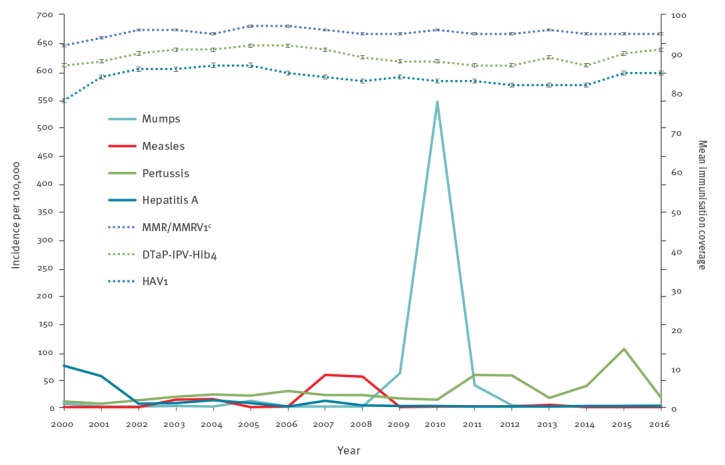
Incidence of selected notifiable vaccine-preventable diseases^a^ and reported overall mean coverage of selected routine childhood vaccinations^b^, Jerusalem district, Israel, 2000–2016

DTaP-IPV-Hib4: diphtheria, tetanus, acellular pertussis, polio, *Haemophilus influenzae* b vaccine, fourth dose; HAV1: hepatitis A vaccine, first dose; MMR/MMRV1: measles-mumps-rubella/measles-mumps-rubella-varicella vaccine, first dose.

^a^ Incidence per 100,000 population of measles, mumps, pertussis and hepatitis A.

^b^ MMR/MMRV1, DTaP-IPV-Hib4 and HAV1, as up-to-date at age 2 years (vaccination completeness).

^c^ MMRV vaccine replaced MMR in 2008.

In the last two decades, several VPD outbreaks emerged in the Jerusalem district (Figure 1). Measles and mumps outbreaks emerged mainly in Jewish ultra-Orthodox communities, with epidemiological links to similar communities in Europe and the United States (US) [10-13]. The district health office’s teams perform surveillance, epidemiological investigations and outbreak control activities; community-wide vaccination campaigns led to outbreaks’ containment, with remarkable population compliance during the campaigns.

Recurrent VPD outbreaks indicated the need for a detailed assessment of vaccination coverage in the affected district. Our study evaluated timeliness and completeness of routine childhood vaccinations in order to identify factors associated with vaccination receipt patterns and to gather information for planning public health intervention programmes.

## Methods

### Setting and study population

The Jerusalem district’s population increased from 1 to 1.2 million between 2009 and 2016 (30% Arabs and 70% Jews; about 40% of the Jews are ultra-Orthodox). Neighbourhoods in the area are homogenous, with Arab, Jewish ultra-Orthodox and Jewish traditional-secular residents. The socioeconomic status of the district’s population is medium to low. The district’s total fertility rate is four (Jewish ultra-Orthodox: 6.2–6.5), compared with three nationally. Children under 6 years comprise 15% of the district’s population [14,15].

The study group for detailed vaccine acceptance evaluation included children born in 2009 in the Jerusalem district. The sample size was calculated taking the following into account: unvaccinated fraction (5–25%), 1.5% precision and a 95% confidence interval (CI). Post adjustment, the study group was selected from the district’s newborns file using the Statistical Package for Social Sciences (SPSS) software v23.0 (IBM, New York, US) random sampling of data procedure (n = 3,180 children, 10.7% of the 29,700 live births registered in the district in 2009). The inclusion criteria were: born in Israel, has a unique identifier (identification (ID) number allowing data matching) and survived 48 months. The exclusion criteria were: born abroad (different schedules), lacks a unique identifier and did not survive 48 months.

### Variables collected

The general variables collected included the child’s date of birth, sex, ethnicity, address, birth order and birthweight, as well as the mother’s age, country of birth and marital status.

#### Vaccination variables

The Jerusalem district routine vaccination coverage is monitored by data aggregation in the up-to-date method (vaccination completeness, Figure 1). The up-to-date method does not reflect vaccination timeliness, which is better assessed by the age-appropriate method (indicating the child’s age at specific vaccine doses). The launch of a national immunisation registry in 2009 enabled appraisal of both completeness and timeliness.

The scheduled immunisations included: hepatitis B vaccine (HBV: at birth, 1 month and 6 months); diphtheria, tetanus, acellular pertussis, polio, *Haemophilus influenzae* b vaccine (DTaP-IPV-Hib: at 2, 4, 6 and 12 months); pneumococcal conjugate vaccine (PCV: at 2, 4 and 12 months); measles-mumps-rubella/measles-mumps-rubella-varicella vaccine (MMR/MMRV: at 12 months) and hepatitis A vaccine (HAV: at 18 and 24 months). MMRV vaccine replaced MMR in 2008 and PCV vaccine was introduced in 2009.

Vaccine doses were defined as valid according to the Israel Ministry of Health (MoH) guidelines for minimum ages and time intervals between doses. Vaccine doses received up to 1 month after the recommended age were considered timely (no delay). The children’s ID numbers and dates of birth were cross-checked against the vaccination registry and vaccination data were extracted. After data assembly, records were available for 3,098/3,180 (97%) of the children. The groups of children with available records and those with missing records had similar birthweight, sex, birth order and maternal variables.

The vaccination data were evaluated at age 24 months (2011) and age 48 months (2013), then re-evaluated at age 7 years (2016). For the present study’s purposes, the following categories were defined according to the child’s vaccination status: (i) age-appropriate (vaccinated at the recommended age or ≤ 1 month later), (ii) mild-moderate delay (delayed ≤ 6 months; mild: > 1 month and ≤ 3 months, moderate: > 3 months and ≤ 6 months) (iii) severe delay (delayed> 6 months) and (iv) unvaccinated (at 48 months).

### Data analysis

Data analysis was performed with SPSS software v23.0. The age-specific immunisation coverage was retrieved from a cumulative fraction of vaccinated children by age and plotted in inverse Kaplan-Meier curves (survival analysis curves). Days of vaccination delay were converted into months as 30.5 days/month. A univariate analysis was performed for each vaccine (HBV3, DTaP-IPV-Hib4, PCV3, MMR/MMRV1, HAV1 and HAV2) exploring child and maternal characteristics for association with vaccination timeliness. Variables with statistical significance at p value < 0.05 were included in the multivariate analysis. A multiple regression analysis model was performed for general variables associated with a child’s vaccination status being up-to-date. Associations between the variables and vaccination status are presented as odds ratio (OR) and 95% CI. A p value of < 0.05 was considered significant for all comparisons.

### Ethical approval

This study was approved by the Israel MoH Institutional Review Board and was conducted according to the relevant MoH instructions. All collected data were treated as confidential, in strict compliance of legislation on observational studies.

## Results

The general characteristics of the 3,098 children born in the Jerusalem district in 2009 and their mothers are presented in Table 1. Half of them (50.7%) were male.

**Table 1:** General characteristics of the study group, children born in 2009 and followed up to 7 years of age, Jerusalem district, Israel, 2016 (n = 3,098)

Variables	n^a^ = 3,098	%
Male	1,571	50.7
Birth weight (g), mean ± SD	3,245 ± 528	NA
Birth weight < 2,500 g	257	8.3
Birth order, mean ± SD	3.4 ± 2.4	NA
Birth order, median (range)	3 (1–14)	NA
Birth order ≥ 4	1,151	37.2
Mother’s age (years), mean ± SD	29.1 ± 6	NA
Mother’s age (years), median (range)	28 (15–55)	NA
Mother’s birth country is Israel	2,506	80.9
Mother’s status is married	3,009	97.1
Maternal education (years), mean ± SD	13.7 ± 2.4	NA
Maternal education, median (range)	14 (4–23)	NA
Ethnicity: Jewish/Arab	2,163/935	70/30
**Month of birth**		
January–March	722	23.3
April–June	762	24.6
July–September	818	26.4
October–December	796	25.7

g: grams; NA: not applicable; SD: standard deviation.

^a^ Unless otherwise specified.

**Figure 2:**
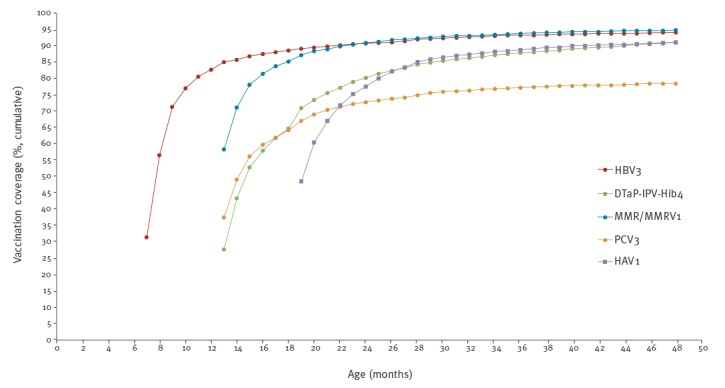
The cumulative proportion of vaccine uptake by child’s age in months using the inverse Kaplan-Meier curves for selected vaccine doses^a^, in children born in 2009 and followed up to 7 years of age, Jerusalem district, Israel, 2016 (n = 3,098)

DTaP-IPV-Hib4: diphtheria, tetanus, acellular pertussis, polio, *Haemophilus influenzae* b vaccine, fourth dose; HAV1: hepatitis A vaccine, first dose; HBV3: hepatitis B vaccine, third dose; MMR/MMRV1: measles-mumps-rubella/measles-mumps-rubella-varicella vaccine, first dose; PCV3: pneumococcal conjugate vaccine, third dose.

^a^ DTaP-IPV-Hib4 is scheduled at 12 months, HAV1 at 18 months, HBV3 at 6 months, MMR/MMRV1 at 12 months (MMRV vaccine replaced MMR in 2008) and PCV3 at 12 months.

The cumulative fraction of vaccine uptake by inverse Kaplan-Meier curves for HBV3, DTaP-IPV-Hib4, MMR/MMRV1, PCV3 and HAV1 are presented in Figure 2. The age-specific rates at three points in time were as follows: for HBV3, 31.5%, 82.8% and 90.8% were vaccinated at 7, 12 and 24 months, respectively. For DTaP-IPV-Hib4, 27.7%, 64.8% and 80.2% at 13, 18 and 24 months. For PCV3, 37.6%, 64.1% and 72.6% at age 13, 18 and 24 months. For MMR/MMRV1, 58.3%, 85.2% and 90.8% at 13, 18 and 24 months. For HAV1, at 19 and 24 months, 48.6% and 78%.

**Figure 3:**
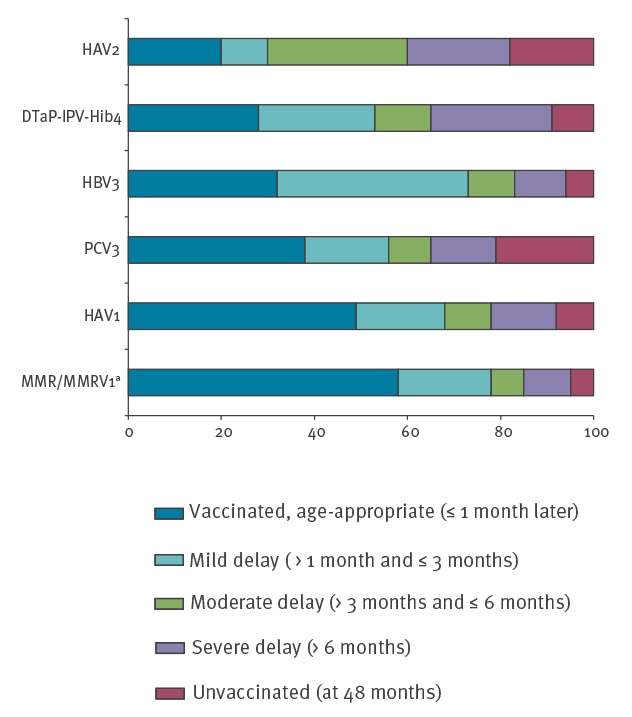
Distribution of vaccination coverage by defined categories (status at the age of 48 months) for selected vaccine doses, in children born in 2009, Jerusalem district, Israel, 2016 (n = 3,098)

DTaP-IPV-Hib4: diphtheria, tetanus, acellular pertussis, polio, *Haemophilus influenzae* b vaccine, fourth dose; HAV1: hepatitis A vaccine, first dose; HAV2: hepatitis A vaccine, second dose; HBV3: hepatitis B vaccine, third dose; MMR/MMRV1: measles-mumps-rubella/measles-mumps-rubella-varicella vaccine, first dose; PCV3: pneumococcal conjugate vaccine, third dose.

^a^ MMRV vaccine replaced MMR in 2008.

The distribution by vaccination categories at age 48 months (Figure 3) showed that, depending on the vaccine, between 82–95% of the children were defined as vaccinated up-to-date. The up-to-date rates for the HBV3, DTaP-IPV-Hib4, PCV3, MMR/MMRV1, HAV1 and HAV2 vaccine doses were 94%, 91%, 79%, 95%, 92% and 82%, respectively. The age-specific vaccination rates showed that only 32%, 28%, 38%, 58%, 49% and 20% of children were defined as age-appropriate for these vaccine doses. The fraction of severe delay was higher in the multiple-dose vaccines (26% for DTaP-IPV-Hib4, 22% for HAV2) compared with 10% for MMR/MMRV1. The fraction of children defined as unvaccinated at 48 months also ranged between 18% for HAV2, 21% for PCV3 (in the cohort year of introduction into the schedule) and 5–9% for the other vaccine doses.

**Figure 4:**
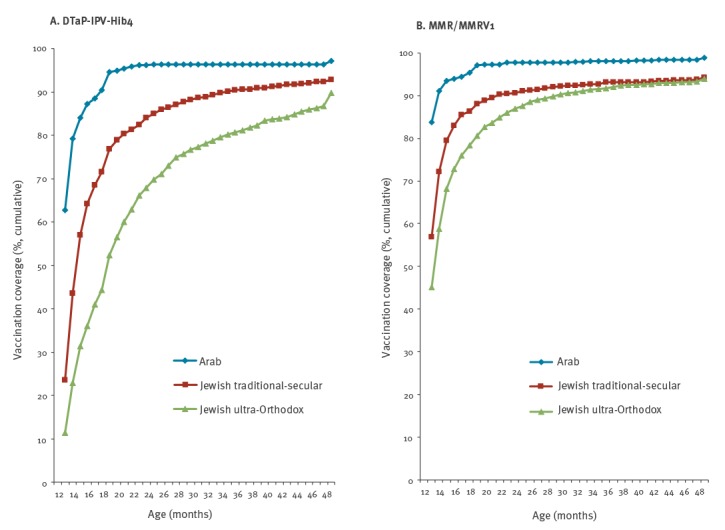
The cumulative proportion of vaccination uptake by age for (A) DTaP-IPV-Hib4 and (B) MMR/MMRV1^a^, in children born in 2009 and followed up to 7 years of age, by main population groups, Jerusalem district, Israel 2016 (n = 3,098)

The cumulative fraction of vaccine uptake among children in the three main population groups—Arab, Jewish ultra-Orthodox and Jewish traditional-secular—for DTaP-IPV-Hib4 and MMR/MMRV1 are presented in Figure 4. Vaccination completeness and timeliness were higher in Arab children compared to Jewish children, with the lowest rates among children in Jewish ultra-Orthodox communities.

DTaP-IPV-Hib4: diphtheria, tetanus, acellular pertussis, polio, *Haemophilus influenzae* b vaccine, fourth dose; MMR/MMRV1: measles-mumps-rubella/measles-mumps-rubella-varicella vaccine, first dose.

^a^ MMRV vaccine replaced MMR in 2008.

Multiple logistic regression analysis with the dependent variable ‘vaccinated up-to-date’ at age 24 months and the study group general variables was performed (Supplement S1). The up-to-date vaccination status defined for age 24 months included the vaccine doses HBV3, DTaP-IPV-Hib4, PCV3 and MMR/MMRV1. The variables that were significantly associated with vaccination delay were: high birth order, ethnicity (higher among Jews vs Arabs), birth in winter (January–March) and delayed receipt of the first dose of DTaP-IPV-Hib vaccine scheduled at age two months (OR: 4.67; 95% CI: 3.72–5.87).

A re-evaluation of vaccination status was carried out for the study group children at the age of 7 years. The vaccination coverage rates showed some increase for all the evaluated vaccine doses: HBV3 (1.7%), DTaP-IPV4 (3.1%), PCV3 (1%), MMR/MMRV1 (1.2%) and HAV2 (8%). The median increase observed in vaccination coverage rates was 2.4%.

## Discussion

The overall childhood vaccination coverage reported in Israel is consistently high [6,7]. While aggregated vaccination rates are high, disaggregated data reveal gaps amid population groups [4,8,16]. Vaccinations gaps and delays, despite appropriate up-to-date coverage, have been reported from developed and developing countries [17-25]. Hence, accurate monitoring of vaccination coverage and timeliness is essential [9,21,26]. The up-to-date vaccination coverage rates were all over 90% for the vaccines evaluated in our study; age-appropriate rates were lower. A similar percentage of children were in the categories ‘mild-moderate delay’ and ‘age-appropriate’ for multiple-dose vaccines. The MMR/MMRV1 vaccine was less delayed, perhaps because its application does not depend on the timing of a previous dose and because efforts are made to ensure timeliness to prevent outbreaks. The 7-valent pneumococcal conjugate vaccine (PCV7) was included into the routine schedule in 2009 and PCV13 replaced PCV7 in 2010 [27]. The coverage rate for the third dose of PCV in our group was lower than the 91% national rate [4]. Mothers in the Jerusalem district reported declining ‘new’ vaccines (e.g. PCV) more often [28]. The reasons for this are unclear and may be attributed to the provision and promotion of new vaccines.

The association between social determinants and health outcomes has been well established [29]. A medium to low socioeconomic status and a high proportion of children in the Jerusalem district have been linked to the spread of communicable disease [10,13]. Delayed vaccinations were associated with a child’s birth order, ethnicity, season of birth and delayed receipt of the first dose of the DTaP-IPV-Hib vaccine. The median child’s birth order was third in Jerusalem, compared with second nationally, with 37% of children born fourth and above. A high birth order has been associated with vaccination delay [18,21-25]. Vaccination completeness and timeliness were higher in Arab children compared to Jewish children in Jerusalem, which is similar to data for the country overall [4,16]. In a polio vaccine campaign in Israel (2013), the compliance was higher in the Arab than in the Jewish population [30]. Birth in the winter months was also associated with childhood vaccination delay. Delays, most of which are unnecessary, are often related to acute respiratory infections during winter [28]. Parents may perceive delaying vaccination as a safer alternative to the routine childhood vaccination schedules [31]. In our group, delayed receipt of the first dose of the DTaP-IPV-Hib vaccine was highly associated with not being up-to-date at 24 and 48 months. In a US survey, children with delayed vaccines at 3 months had significantly lower up-to-date coverage (at 19–35 months) compared to children without early delay [32]. In a study among Jewish ultra-Orthodox mothers in Israel, infant vaccination receipt at age 2 months was highly predictive for later adherence to the schedule [33]. Children in Jewish ultra-Orthodox communities were found at risk for delayed and missing vaccinations [10,13], as in these communities delay was not perceived as affecting a child’s health [28].

The results of this study are subject to limitations. Only children with full data were included and this may have led to an underestimate of vaccine delay. However, counting documented vaccine doses was the only way to obtain accurate vaccination dates. Yet, even the digital records may have been incomplete, with some dates not registered, resulting in an overestimate of vaccine delay. Children who left the area or died were also excluded; therefore, there may be a bias in estimates of the cumulative proportion of vaccination. As for factors affecting vaccination receipt, we included mainly sociodemographic factors. We were unable to include health-related parameters or factors related to the performance of the preventive health services supplier, which should be further evaluated.

After the completion of our study, from March to December 2018, measles importation to Israel resulted in spread to unvaccinated persons, with some 3,150 notified cases (https://www.health.gov.il/English/Topics/Pregnancy/Vaccination_of_infants/Pages/measles.aspx). The outbreak reached Jerusalem in late August 2018 and at present some 1,800 cases have been notified in Jerusalem; 82% (1,470) are children under 15 years of age who almost exclusively reside in ultra-Orthodox Jewish neighbourhoods. An 18-month-old toddler died in Jerusalem in November 2018 and in December 2018 an 82-year-old woman in Jerusalem became the second fatality from the outbreak. The child’s death was the first recorded death from measles in Israel in 15 years

The recent outbreak and our findings denote the importance of accurate vaccination data for detecting risk groups, reducing missed opportunities and planning tailored immunisation programmes. Vaccination delay is a common phenomenon that may induce pockets of susceptible populations to VPD outbreaks; therefore, it should be adequately addressed within the vaccine hesitancy spectrum. Vaccine hesitancy refers to the delay in acceptance or refusal of vaccines despite availability of vaccination services. It includes factors such as complacency, convenience and confidence [34]. It has been estimated that 7.5–9% of Israeli parents deviate from the routine vaccination schedule mostly as a consequence of parental decision [35,36]. While most Israeli parents (90%) reported that they had fully immunised their children, the confidence in official recommendations declined from 87% in 2008 to 72% in 2016 [37]. Addressing various forms of vaccine hesitancy is an increasingly complex challenge for health professionals [34,38]. The preventive framework should combine vaccination plans with health promotion measures, the most effective of which are multi-component [34,39]. Particularly in areas and communities with suboptimal vaccine uptake (as found in our study), efforts and budget allocations should prioritise investments that support availability, accessibility and appropriateness of preventive services for children [40]. Implementation of systematic supplementary immunization activities (SIAs) such as mass vaccination campaigns is still essential while the current preventive services are being strengthened [41]. The integration of multiple vaccination-related activities will hopefully further reduce the burden of vaccine-preventable diseases in children.

## Supplementary Data

449000 Supplement S1
