# Supplementary material for: Timeliness and completeness of routine childhood vaccinations in young children residing in a district with recurrent vaccine-preventable disease outbreaks, Jerusalem, Israel
Source: Euro Surveill. 2019 Feb 7;24(6):1800004. doi: 10.2807/1560-7917.ES.2019.24.6.1800004 (PMC6373067; doi:10.2807/1560-7917.ES.2019.24.6.1800004)
Supplement: Supplement S1 [file 180004_STEIN-ZAMIR_Supplement.pdf]

This supplementary material is hosted by *Eurosurveillance* as supporting information alongside the article ‘Timeliness and completeness of routine childhood vaccinations in young children residing in a district with recurrent vaccine-preventable disease outbreaks, Jerusalem, Israel’ on behalf of the authors, who remain responsible for the accuracy and appropriateness of the content. The same standards for ethics, copyright, attributions and permissions as for the article apply. *Eurosurveillance* is not responsible for the maintenance of any links or email addresses provided therein.

Table 2. Multiple logistic regression - dependent variable “vaccinated up-to-date” at age 24 months

Variable	Adjusted Odds Ratio (95% CI)	P value
**Sex**		
**Female**	1	
**Male**	0.88 (0.75 – 1.04)	NS
**Birth weight**		
**Birth Weight ≥ 2500 gr**	1	
**Birth Weight <2500 gr**	0.67 (0.44 – 1.03)	NS
**Ethnicity**		
**Arabs**	1	
**Jews Traditional Secular**	1.38 (1.1 – 1.7)	0.06
**Jews Ultra-Orthodox**	1.55 (1.23 – 1.93)	0.0001
**Birth order**		
**Birth order 1-3**	1	
**Birth order ≥ 4**	1.46 (1.22 – 1.73)	0.0001
**Mother's status**		
**Married**	1	
**Not married**	1.16 (0.71 – 1.88)	NS
**Month of birth**		
**January-March**	1.4 (1.1 – 1.76)	0.004
**April-June**	1.15 (0.92 – 1.45)	NS
**July-September**	0.91 (0.73 – 1.15)	NS
**October-December**	1	
**DTaP first dose**		
**Timely**	1	
**Delayed**	4.67 (3.72 – 5.87)	0.0001
